# Evaluation of Two Particle Number (PN) Counters with Different Test Protocols for the Periodic Technical Inspection (PTI) of Gasoline Vehicles

**DOI:** 10.3390/s24206509

**Published:** 2024-10-10

**Authors:** Anastasios Melas, Jacopo Franzetti, Ricardo Suarez-Bertoa, Barouch Giechaskiel

**Affiliations:** 1 European Commission, Joint Research Centre (JRC), 21027 Ispra, Italy; jacopo.franzetti@ec.europa.eu (J.F.); ricardo.suarez-bertoa@ec.europa.eu (R.S.-B.); 2 Mining and Energy Engineering School, Universidad Politécnica de Madrid, c/Rios Rosas 21, 28003 Madrid, Spain

**Keywords:** in-use vehicle emissions, petrol car, GDI, PFI, PN-PTI, diffusion charger, condensation particle counter, sub-23 nm particles

## Abstract

Thousands of particle number (PN) counters have been introduced to the European market, following the implementation of PN tests during the periodic technical inspection (PTI) of diesel vehicles equipped with particulate filters. Expanding the PN-PTI test to gasoline vehicles may face several challenges due to the different exhaust aerosol characteristics. In this study, two PN-PTI instruments, type-examined for diesel vehicles, measured fifteen petrol passenger cars with different test protocols: low and high idling, with or without additional load, and sharp accelerations. The instruments, one based on diffusion charging and the other on condensation particle counting, demonstrated good linearity compared to the reference instrumentation with R-squared values of 0.93 and 0.92, respectively. However, in a considerable number of tests, they registered higher particle concentrations due to the presence of high concentrations below their theoretical 23 nm cut-off size. The evaluation of the different test protocols showed that gasoline direct injection engine vehicles without particulate filters (GPFs) generally emitted an order of magnitude or higher PN compared to those with GPFs. However, high variations in concentration levels were observed for each vehicle. Port-fuel injection vehicles without GPFs mostly emitted PN concentrations near the lower detection limit of the PN-PTI instruments.

## 1. Introduction

Airborne particle counters have a wide area of research and industrial applications [1], such as clean room measurements in the pharmaceutical industry [2] and nanoparticle synthesis with aerosol reactors [3]. During and after the COVID-19 pandemic, the use of particle counters for face mask filtration efficiency measurements has also increased [4,5]. Another widespread application of particle counters is indoor [6] and outdoor [7] air quality monitoring. Clinical studies have shown that particulate matter PM2.5 (airborne particles smaller than 2.5 μm) is harmful to human health [8,9], while numerous studies suggest that sub-micrometer particles and especially ultrafine particles (<100 nm size) can cause adverse health effects [10,11] and impact climate change [12]. In this context, vehicles with combustion engines, a well-known source of ultrafine particles, are subject to a particle number limit for sizes down to 23 nm in the European Union (EU) during their type approval (permission to circulate in the EU) [13]. Recently, a particle number (PN) limit down to 23 nm sizes was introduced during the periodic technical inspection (PTI) of diesel vehicles circulating in four European countries [14,15,16]. Currently, tens of thousands of PN counters are used in the inspection centers of these countries for PN-PTI measurements [17].

Particle number counters for PTI consist of a sampling probe, a sampling line (optional), a sample precondition device that dilutes and/or removes volatile particles, and a particle detection device. For PTI applications, the instruments should be compact, robust, and equipped with a series of self-controls that allow non-experts to identify if they operate correctly. Moreover, their cost should be low to fulfill the needs of the inspection centers. All PN-PTI instruments circulating in the market should be type-examined and meet metrological and technical requirements that include specifications for maximum permissible error, linearity, and repeatability but also the ability to handle disturbances like a mechanical shock. Additionally, these instruments should operate in a wide range of ambient conditions that could be encountered in inspection centers. PN-PTI instrument requirements in different European countries were analytically presented elsewhere [18,19]. In March 2023, the European Commission published harmonized requirements in a recommendation on PN measurement of diesel vehicles at their PTI [20].

Particle sensors of PN-PTI instruments are based on two different measurement technologies: condensation particle counting (CPC) [21] and diffusion charging (DC) [22]. CPCs are also used for PN counting during vehicle type approval in laboratory and on-road [13]. They employ a working fluid (typically isopropanol for PN-PTI applications) which, after evaporating, is mixed with aerosol particles in a saturator. The saturated flow then passes through a condenser, and droplets grow to sizes that can be detected by light scattering. This method allows for precise single-particle counting, up to a number concentration that varies according to the CPC design. The cut-off size of CPCs mainly depends on the temperature difference between the saturator and the condenser. For automotive applications, the cut-off size of CPCs has been typically 23 nm [13] and lately 10 nm [23] but CPCs can measure even in the sub-10 nm size range [24]. Previous research has shown that 23 nm CPCs counting efficiency has a strong material dependence [25,26].

DCs charge particles using a corona charger and measure the electrical current of particles, which depends on the number concentration [27] but also on particle size, morphology [18,28,29], and pre-charge [30,31]. Deficiencies in DCs have been mitigated with the implementation of advanced designs that replaced the single-stage DCs (one current measurement stage). Nonetheless, they are still available in the market, especially for PN-PTI applications [18,19]. Some examples of advanced designs include two-stage DCs (implementation of a second aerosol measurement stage; a diffusion and a filter stage) that reduce size dependence [32], DCs with induced currents and substitution of the typically used aerosol electrometer with a Faraday cage [33], and DCs operation at reduced pressure conditions [34]. DCs with a cut-off at 23 nm are used for on-road measurement tests both for light- and heavy-duty vehicles [35,36] and are also very common for PN-PTI applications [19,37,38]. DCs capable of measuring down to 10 nm have recently been developed [39].

At present, PN-PTI measurements are required only for diesel vehicles. The test is conducted at low idling and is based on several scientific studies showing the efficiency and robustness of this method to detect high PN emitters as well as demonstrating a linear correlation between low idling PN concentrations and type approval PN emissions [14,40,41]. Gasoline vehicles are also a source of ultrafine particles [42,43,44]. Those equipped with a gasoline direct injection (GDI) engine are subject to a PN limit at type approval starting from Euro 6 while vehicles with port-fuel injection (PFI) engines will have a PN limit in Euro 7 [13]. Modern GDI vehicles in Europe are equipped with gasoline particulate filters (GPFs) [45]. Contrary to diesel vehicles, no correlation has been found between type approval and low idling emissions of these vehicles [40]. Developing a meaningful PN-PTI test procedure for petrol cars (both GDI and PFI engines) has proved to be challenging.

In addition to the measurement procedure, there are other challenges related to the PN-PTI instruments used for petrol vehicles. Previous studies have shown that both DC- and CPC-based PN-PTI instruments exhibit larger deviations compared to reference instrumentation when a significant portion of the aerosol particles falls within the sub-23 nm size range [14,37]. This is particularly relevant to petrol vehicles, as GDI and PFI engines may emit smaller particles compared to diesel engines, with a large fraction lying in the sub-23 nm range [23,46,47]. Moreover, petrol engine exhaust has considerably higher humidity compared to diesel engine exhaust, which can affect the operation of PN-PTI instruments (e.g., through condensation) and their sensor technologies and specifically CPCs [48,49] and DCs (e.g., by affecting the ion properties or impacting the electrometers [50,51,52]). Another challenge for the counters may be related to possible exhaust pressure changes taking place during potential test procedures and that could impact the dilution ratio of some diluter technologies [53]. An example of test procedures that could result in pressure exhaust changes could be a combination of low and high idling testing, similar to the CO testing procedure for petrol cars currently in place at PTI stations [54]. Previous research has examined the performance of PN-PTI instruments when measuring diesel vehicles [37,55,56] but only a few studies have challenged these instruments with petrol vehicles, which were limited in scope [14,57].

The objective of this study is to provide insights into PN-PTI testing of gasoline vehicles. This study presents, for the first time, an assessment of the performance of two PN-PTI instruments, originally designed for diesel vehicle testing, when measuring gasoline vehicles equipped with GDI and PFI engines. For this purpose, the exhaust solid particle number of fifteen light-duty vehicles with and without GPFs is measured with two PN-PTI devices based on CPC and DC technology, and the results are compared to reference instrumentation employed for PN measurement during vehicle type approval. Additionally, particle number measurements down to 10 nm in size are conducted to investigate the impact of particles below the lower diesel PN-PTI size limit of 23 nm. Five different test protocols are examined. Finally, the overall performance of the PN-PTI instruments is evaluated, and insights are provided regarding sources of deviation from the reference instrumentation.

## 2. Materials and Methods

The tests were conducted in the vehicle emissions laboratory of the Joint Research Centre (JRC) of the European Commission in Ispra, Italy. The temperature was maintained at 20 ± 3 °C across all experiments.

### 2.1. PN-PTI Instruments

Two PN-PTI instruments were used in this study; one was based on diffusion-charging, manufactured by AVL DiTEST (Graz, Austria), and the other on condensation particle counting and it was the NPET of TSI (Shoreview, MN, USA). Both devices measure solid particle number concentration down to 23 nm size and are widely used for PN-PTI testing of diesel vehicles. The AVL DiTEST counter used in this study underwent type-examination in Germany (for more information on the instrument requirements during type-examination refer to [58]) with the same or similar versions used for PN-PTI in Switzerland, Belgium, and the Netherlands. The TSI NPET was the first instrument designed for PN-PTI [41], is type-examined in Switzerland [59], and is often used as a reference instrument [18,56]. Counting efficiency tests are performed with monodisperse particles in Germany and polydisperse particles in Switzerland. Linearity is checked with polydisperse soot particles and the maximum permissible error in Germany is 25% in the concentration range 5 × 10^3^ #/cm^3^–5 × 10^5^ #/cm^3^ and in Switzerland 30% in the concentration range 5 × 10^4^ #/cm^4^–5 × 10^6^ #/cm^3^. In the following, the AVL DiTEST counter will be referred to as PTI_23_-DC and the TSI counter as PTI_23_-CPC.

The PTI_23_-DC comprises a sampling probe that is introduced in the tailpipe, a non-heated sampling line with a length of ~4 m, a water trap to remove exhaust humidity, a volatile particle remover (VPR) based on an evaporation tube at 150 °C, and a PN counter. The PN counter is a pulsed mode diffusion charger composed of three elements: (i) a constant unipolar corona charger, (ii) a pulsed precipitator, and (iii) a Faraday cage [38]. PTI_23_-DC is portable, weighs 8.6 kg (without considering the sampling probe), and can measure PN concentrations up to 10^7^ #/cm^3^. Similar to other diffusion chargers, its efficiency depends on the morphology of particles. One study reported lower efficiencies when measuring NaCl particles compared to CAST-generated particles [38], while another found higher counting efficiencies when measuring spark-generated graphite particles [18]. Smaller differences can be observed when measuring various CAST device particles [60]. These differences between calibration materials can be corrected by applying calibration setup correction factors [18,19].

The PTI_23_-CPC dilutes the exhaust near the sampling point, contains a VPR based on a catalytic stripper at 350 °C [61], and measures particle number concentration using a CPC that operates with isopropanol. The sampling line is ~4 m. PTI_23_-CPC is portable and weighs 15 kg, including the sampling line. It can measure PN concentrations up to 5 × 10^6^ #/cm^3^ but the prototype version used in this study could measure up to 1 × 10^7^ #/cm^3^. Previous studies have shown that its counting efficiency is not influenced by particle materials such as soot, salt, or silver [18,19,60]. It can operate in a wide range of temperatures and altitudes.

Both PN-PTI counters used in this study were calibrated within one year prior to the testing campaign. The PTI_23_-DC was sent to the manufacturer after the testing campaign and it was found to overestimate particle concentration by 32%. All PTI_23_-DC results presented in this paper, in accordance with the manufacturer’s request, were divided by a factor of 1.32.

### 2.2. Reference Instrument

The reference PN instrument was the Advanced Particle Counter (APC xApp) from AVL (Graz, Austria). The APC consists of a volatile particle remover with a hot and a cold dilution stage that uses a catalytic stripper at 350 °C to remove volatiles, a CPC with a cut-off at 23 nm (AVL), and a CPC with a cut-off at 10 nm (AVL). Additionally, upstream of the APC, a heated sampling line at 110 °C was used to prevent water condensation. The instrument was calibrated less than a year prior to the testing campaign. The APC is employed for vehicles’ laboratory type approval particle number measurements. Accordingly, it complies with a ±5% maximum error during linearity tests. Its dilution factors are adjustable by the user and it can measure in the order of 10^7^ #/cm^3^ (and even higher). In our study, for the PTI measurements, the device was set to raw exhaust mode. From now on, the solid particle number (SPN) concentration measured with the APC will be reported as LAB_23_ for the CPC with a cut-off at 23 nm and LAB_10_ for the CPC with a cut-off at 10 nm. Reference instruments, LAB_23_ and LAB_10_, were calibrated within one year prior to the testing campaign.

### 2.3. Experimental Setup

**Figure 1:**
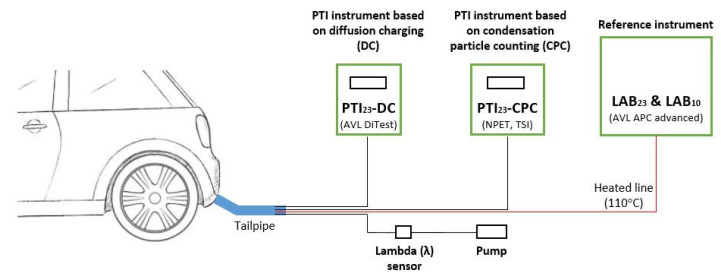
Schematic of the experimental setup. With red, we show heated lines. PTI = periodic technical inspection.

Figure 1 shows a schematic of the experimental setup. PN emissions from different petrol cars were measured at the tailpipe by the two PN-PTI instruments and the reference device. A lambda (λ) sensor was employed to monitor the air–fuel equivalence ratio. The flow for λ measurements was extracted using a pump. The exhaust flow not used for measurements was directed to ventilation. Sampling probes were inserted at a depth of at least 20 cm in the exhaust pipe.

At the start of each day, a sequence of checks was conducted to ensure the proper functioning of each device. LAB_23_ and LAB_10_ automatically performed checks required by the Worldwide Harmonized Light Vehicles Test Procedure (WLTP), such as the CPC zero concentration and inlet flow checks [62]. In accordance with PTI requirements, the PTI_23_-DC underwent a series of self-diagnostic checks, including leak and zero checks. In contrast, the PTI_23_-CPC was a prototype device and did not have automatic controls. The zero of the CPC-based devices was checked manually at the beginning of each testing day with a high-efficiency particulate air (HEPA) filter.

During the testing campaign, the instruments did not exhibit any issues related to exhaust water and humidity. Furthermore, no error messages were observed for any other malfunctions.

### 2.4. Test Procedure

Currently, there is no standard PN-PTI test procedure for gasoline vehicles. Five different PN-PTI test protocols were used for the PN-PTI instruments evaluation that covered different possibilities for static tests.

**Figure 2:**
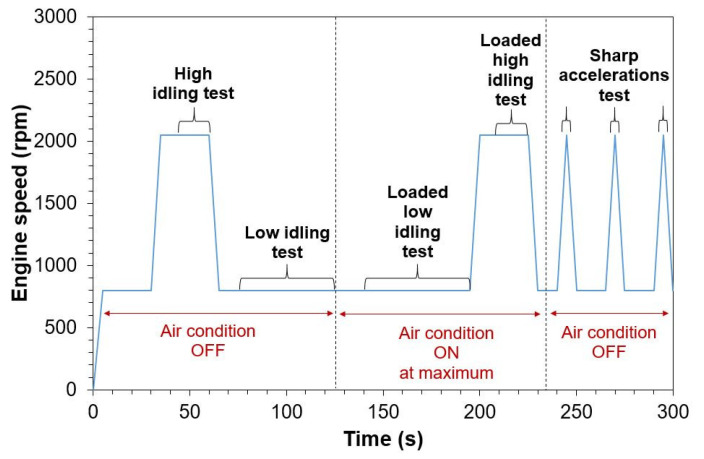
Test procedure followed in this study including five different tests with a static vehicle. The plotted engine speed is indicative. At high idling (with or without load) and at sharp accelerations, the maximum engine speed was between 2000 and 3000 rpm. The brackets show the fraction of each test that was taken into account for the solid particle number average. For each sharp acceleration, the maximum concentration of each repetition was considered as the test emissions.

Vehicles were fueled with market gasoline [63] and were tested with hot engines, meaning with engine coolant temperature > 70 °C. The SPN measurement devices registered data during the entire test. Figure 2 illustrates an example of the test procedure. The first 30 s after the ignition of the thermal engine was considered a stabilization period. Afterward, five different test procedures were conducted: low and loaded low idling, high (2000–3000 rpm) and loaded high idling, and finally three sharp accelerations reaching up to 2000 and 3000 rpm. The load was created by activating the air conditioning system of the vehicles at maximum. The tests were performed at the subsequent sequence: high idling, low idling, loaded low idling, loaded high idling, and sharp accelerations. The total duration of the full sequence from the engine ignition was 300 s. For each test, only a portion was taken into consideration when determining the average SPN concentration. Specifically, the first 10 to 15 s were considered as stabilization time periods (e.g., when accelerating from low idling to ~2000 rpm), and the rest was used for determining the average SPN emissions for each test. For the ‘sharp accelerations’ tests, the maximum SPN for each acceleration was recorded. It should be recalled that the protocol for diesel engines includes a 15 s stabilization period and at least a 15 s measurement at low idling.

### 2.5. Vehicles

Fifteen light-duty petrol vehicles were used in this study (Table 1). Ten of them had a GDI engine and the remaining five had a PFI engine. Among the GDI vehicles, five were equipped with GPFs. The Euro classifications ranged from Euro 5b to Euro 6d. It is important to note that there is no SPN limit during type approval for PFI vehicles, while for Euro 6b GDI vehicles, the limit is 6 × 10^12^ #/km, and for more recent GDI vehicles (Euro 6c, Euro 6d, and Euro 6e), it is 6 × 10^11^ #/km.

## 3. Results and Discussion

### 3.1. Examples of Real-Time Concentrations

**Figure 3:**
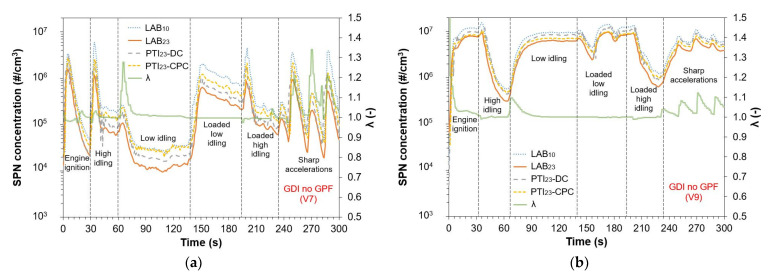
Particle number concentrations and lambda values during testing at high, low, loaded low, loaded high idling, and sharp accelerations with a stationary vehicle for two gasoline direct injection (GDI) vehicles without gasoline particulate filters (GPFs): (**a**) V7; (**b**) V9.

Figure 3 displays the SPN emissions of V7 (Figure 3a) and V9 (Figure 3b) measured using the two PTI instruments, PTI_23_-DC and PTI_23_-CPC, and the reference instruments, LAB_23_ and LAB_10_. The secondary *y*-axis shows lambda values. Both V7 and V9 are Euro 6b GDI vehicles without GPFs.

After engine ignition, LAB_23_ emissions were high (>10^6^ #/cm^3^) for both V7 and V9. For V7, these emissions significantly decreased after ~15 s (<10^5^ #/cm^3^), while for V9, they remained at high levels (>10^6^ #/cm^3^). For V7, during engine acceleration to high idling, LAB_23_ increased sharply and subsequently decreased and stabilized. For V9 the high engine ignition emission decreased. The stabilized LAB_23_ values were 9.7 × 10^4^ #/cm^3^ for V7 and 6.1 × 10^5^ #/cm^3^ for V9. At low idling, LAB_23_ decreased significantly for V7 while the opposite trend was observed for V9. Specifically, for V7, LAB_23_ concentration was 1.5 × 10^4^ #/cm^3^, and for V9, 6.2 × 10^6^ #/cm^3^. Note that the low idling limit for diesel vehicles is 2.5 × 10^5^ #/cm^3^. At loaded low idling LAB_23_ of V7 increased (3.8 × 10^5^ #/cm^3^) compared to low idling, while V9 was not significantly influenced (7.8 × 10^6^ #/cm^3^). Loaded high idling emissions were higher than high idling. For V7, LAB_23_ was 1.4 × 10^5^ #/cm^3^, and for V9, it increased to 1 × 10^6^ #/cm^3^. At the three sharp accelerations, the maximum LAB_23_ concentrations varied significantly for V7, 2.5 × 10^5^–1.2 × 10^6^ #/cm^3^, while for V9, they were more stable 5.1 × 10^6^–6.9 × 10^6^ #/cm^3^. LAB_10_ was in almost all tests for V7 more than two times higher than LAB_23_. For V9, LAB_10_ was ≥50% showing that the 2 vehicles emitted high particle concentrations below the cut-off size of 23 nm. During low, loaded low, high, and loaded high idling tests, the lambda values ranged from 1 to 1.01. Note that lambda limits at high idling during PTI are 0.97–1.03. At sharp accelerations, the maximum lambda ranged from 1.1 to 1.3.

The PN-PTI instruments measured higher than LAB_23_ at all tests. PTI_23_-DC was in good agreement with LAB_23_ when measuring V7, with differences of ≤13% in most cases. Only at low idling did PTI_23_-DC measure 32% higher than LAB_23_, and at one sharp acceleration, it measured 45% higher. To put the results in perspective, the German PN-PTI regulation defines a maximum permissible error of 50% when measuring vehicle exhaust emission with PN-PTI instruments. For V9, the differences between PTI_23_-DC and LAB_23_ were higher, ranging from 23% to 81%, but for the sharp accelerations, the agreement was excellent (≤4% differences). PTI_23_-CPC significantly overestimated the emissions of V7, measuring 44–99% higher compared to LAB_23_. For V9, the agreement was much better, with differences ranging from 8% to 48%.

### 3.2. Comparison of PTI with Reference Instruments

**Figure 4:**
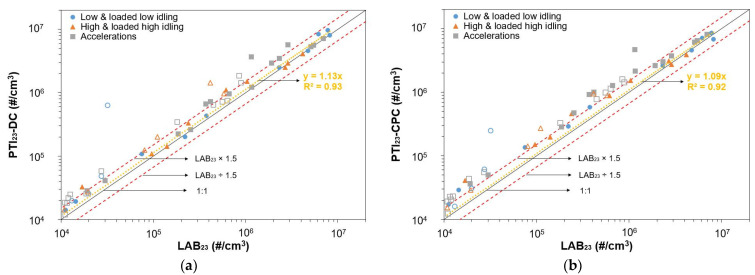
Particle number concentrations measured by the PTI instruments (**a**) PTI_23_-DC and (**b**) PTI_23_-CPC, against the reference LAB_23_. Solid markers represent vehicles with gasoline direct injection (GDI) engines and markers with no fill represent vehicles with port-fuel injection (PFI) engines. The solid black line represents a 1:1 relation. The dashed red lines represent 1.5 times LAB_23_ and LAB_23_ divided by 1.5. The dotted yellow line is a linear fit of the entire data (all plotted tests fitted) with the intercept set to zero.

Figure 4 shows the PN emission measurements of the fifteen vehicles listed in Table 1, as obtained with PTI_23_-DC (Figure 4a) and PTI_23_-CPC (Figure 4b), plotted against LAB_23_. For each vehicle, 3 points are included (low and loaded low idle, high and loaded high idle, accelerations) with a total of 57 points. Only concentrations in the range 10^4^–10^7^ #/cm^3^ are presented and fitted to ensure that concentrations are well within the measurement range of the PTI instruments. Vehicles V1 to V4 emitted in all tests PN concentrations lower than 10^4^ #/cm^3^ and are not included. GDI vehicles are represented with markers with solid fill and PFI vehicles without fill. The dotted yellow line represents a linear fit of the entire dataset (all three subsets) with an intercept of zero. The black line shows a 1:1 PTI device and LAB_23_ relation, while the red lines serve as a visual guide, representing 1.5 times the LAB_23_ value and LAB_23_ divided by 1.5.

The linearity of both PTI devices was very good, with R^2^ values of 0.93 for PTI_23_-DC and 0.92 for PTI_23_-CPC, determined mainly by the high concentration points. The slope of the linear fit was near unity, at 1.13 for the PTI_23_-DC and 1.09 for the PTI_23_-CPC. In most cases, the two instruments measured higher values than the reference LAB_23_. The difference was greater than 50% in 22 tests for PTI_23_-DC and in 36 tests for PTI_23_-CPC. Interestingly, the differences were much higher when measuring PFI vehicles, with 65% of the cases for PTI_23_-DC and 87% for PTI_23_-CPC exhibiting differences greater than 50%. The ratio of differences greater than 50% for GDI vehicles was 21% for PTI_23_-DC and 47% for PTI_23_-CPC. The higher deviation for PFI vehicles may be related to smaller particle sizes emitted by these cars. Another interesting observation is that proportionally more tests with high differences were observed when the engine operated at high rpm (high and loaded high idling and sharp accelerations).

### 3.3. Sub-23 nm Particles

**Figure 5:**
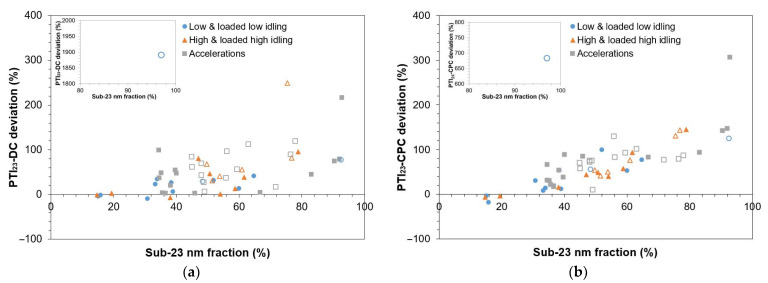
The deviation (%) of the PTI instruments (**a**) PTI_23_-DC and (**b**) PTI_23_-CPC, with respect to sub-23 nm fraction (%). Markers with solid fill represent vehicles powered with gasoline direct injection (GDI) engines and markers without fill represent vehicles with port-fuel injection (PFI) engines. In the figure, the inset displays a deviation that is much higher than the corresponding *y*-axis values.

In this subsection, we investigate the effect of sub-23 nm particle fractions on PTI instrument discrepancies. The sub-23 nm fraction is defined as (LAB_10_ − LAB_23_)/LAB_10_. Figure 5a plots the PTI_23_-DC deviation, which is defined as (PTI_23_-DC − LAB_23_)/LAB_23_, against the sub-23 nm fraction. Figure 5b similarly plots the PTI_23_-CPC deviation. With solid fill symbols, we plot GDI vehicles and without fill symbols PFI vehicles. In most cases, the sub-23 nm fraction was greater than or equal to 30%, corresponding to geometric mean diameters (GMDs) less than or equal to 35 nm, assuming a unimodal particle size distribution with σg ~1.7 [64]. PFI vehicles emitted more sub-23 nm particles compared to GDI vehicles, with an average fraction of 60% for PFI vehicles and 47% for GDI vehicles. Furthermore, among various tests, we observed varying sub-23 nm fractions. GDI vehicles at low idling emitted on average sub-23 nm fraction of 38%, while at high rpm (i.e., at high, loaded high idling and at sharp accelerations) the fraction was 51%. It was not possible to evaluate whether these high concentrations of particles in the range of 10–23 nm emitted during the PN-PTI tests occur also at type approval testing. Note that diesel vehicles emit high sub-23 nm particle concentrations at low idling but no proportionality with type approval was found [14].

Figure 5 shows that as the sub-23 nm fraction increases, the deviation between the PTI instruments and LAB_23_ also increases. The higher sub-23 nm fraction of PFI vehicles as well as at tests performed at high rpm may be the rationale for the higher deviations observed in Figure 4. PTI instruments measure higher values than LAB_23_. Similar results were observed also in previous studies on diesel vehicles [14,37]. A possible reason for this is that PTI instruments have higher counting efficiency below the cut-off size of 23 nm compared to LAB_23_. This difference may originate from different calibration processes: the PTI devices are calibrated together with the sample preconditioning unit, while for LAB_23_, the CPC is calibrated and the VPR losses are corrected with a particle concentration reduction factor (PCRF), which is an average of PCRFs at 100 nm, 50 nm, and 30 nm, and may underestimate particle losses at smaller sizes. Furthermore, the detection efficiency of the reference instrument drops more sharply at 23 nm.

PTI_23_-DC deviations increase with sub-23 nm fractions but have a wider spread compared to PTI_23_-CPC deviations, which correlate better with the presence of nucleation particles. For sub-23 nm fractions higher than 60%, which would correspond to a GMD of approximately 22 nm, in almost all tests, deviations are greater than 50%. In one case, for a PFI vehicle (V11) at loaded low idling, PTI_23_-DC measures approximately 1900% higher than LAB_23_ and PTI_23_-CPC approximately 700% higher. In absolute levels, the PTI_23_-DC measured ~6 × 10^5^ #/cm^3^ more than LAB_23_ and the PTI_23_-CPC 2.2 × 10^5^ #/cm^3^. These high deviations were observed for a sub-23 nm fraction of 97%, meaning that GMD was well below 23 nm. This high deviation is plotted in a separate inset within Figure 5. The average deviation of PTI_23_-DC and PTI_23_-CPC compared to LAB_23_, excluding the outlier of V11, was 48% and 67%, respectively.

### 3.4. Summary of PN-PTI Test Protocol Results

**Figure 6:**
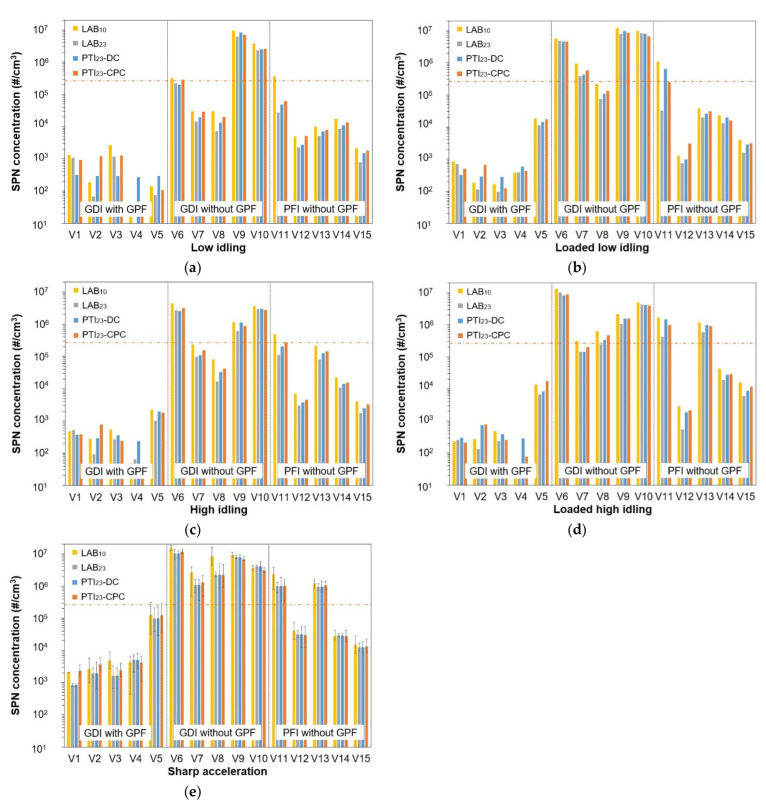
Summary of PN-PTI instruments and the reference LAB_23_ and LAB_10_ measurements using five different test protocols: (**a**) low idling; (**b**) loaded low idling; (**c**) high idling; (**d**) loaded high idling; and (**e**) sharp accelerations. The horizontal dotted red line indicates the diesel vehicles’ PN-PTI limit of 250,000 #/cm^3^ proposed in the European Commission guidelines. In panel 6e, the average of three sharp accelerations is plotted and the bars show the highest and the lowest measured SPN values. GDI = Gasoline Direct Injection. PFI = Port-Fuel Injection. GPF = Gasoline Particulate Filter.

Figure 6 summarizes the SPN concentrations emitted by the 15 tested vehicles. Figure 6a plots the low idling results, Figure 6b the loaded low idling, Figure 6c the high idling, Figure 6d the loaded high idling, and Figure 6e the average of the peak SPN emissions during the three sharp accelerations. The horizontal dotted red line indicates the current PN-PTI limit of 250,000 #/cm^3^ proposed in the Commission guidelines for diesel vehicles. The two vertical solid black lines divide each figure into three parts: the first on the left shows the test results for GDI vehicles with GPFs, the second for GDI vehicles without GPFs, and the third for PFI vehicles. In Figure 6e, we present the average of the three sharp accelerations and the bars indicate the highest and lowest registered concentration values.

GDI vehicles equipped with GPFs tested using the reference LAB_23_ and LAB_10_ emitted SPN concentrations below 10^4^ #/cm^3^ in nearly all protocols. Only V5 exceeded 10^4^ #/cm^3^ at loaded low idling, at loaded high idling, and at sharp accelerations. The SPN concentrations (both down to 23 and 10 nm) of V5 at sharp accelerations exceeded 10^5^ #/cm^3^ and in one case even reached 2.5 × 10^5^ #/cm^3^, which is the diesel vehicles limit. V5 had a GPF but it was not assessed with the type approval WLTC for its effectiveness.

GDI vehicles without GPFs showed varying SPN emissions across different tests. Vehicles V6 to V8 had higher concentrations at high idling compared to low, while V9 and V10 showed the opposite trend. The different behavior of GDI vehicles at the different test protocols shows that a combination of tests cannot be excluded. Loaded tests increased SPN emissions for all GDI vehicles without GPFs. At low idling, concentrations of V6 to V10 as measured with LAB_23_ were in the range of 7 × 10^3^–6 × 10^6^ #/cm^3^, at loaded low idling 8 × 10^4^–8 × 10^6^ #/cm^3^_,_ at high idling 10^4^–3 × 10^6^ #/cm^3^, at loaded high idling 10^5^–8 × 10^6^ #/cm^3^, and the lower peak during sharp accelerations was 2.8 × 10^5^ #/cm^3^ while the average of the peaks was 5 × 10^6^ #/cm^3^.

LAB_10_ measured on average twice the concentrations of LAB_23_ for vehicles without GPFs, except for V8 during sharp accelerations, where LAB_10_ was even 10 times higher than LAB_23_. At low idling, LAB_10_ measured concentrations in the range 3 × 10^4^–9 × 10^6^ #/cm^3^, at loaded low idling 2 × 10^5^–1.2 × 10^7^ #/cm^3^, at high idling 8 × 10^4^–4 × 10^6^ #/cm^3^, at loaded high idling 3 × 10^5^–10^7^ #/cm^3^, and the lower peak during sharp accelerations was 4.7 × 10^5^ #/cm^3^ while the average of the peaks was 8 × 10^6^ #/cm^3^.

As expected, GDI vehicles with GPFs had lower concentrations than vehicles without GPFs. The ratio of the minimum SPN concentrations measured with LAB_23_ for GDI vehicles without GPFs to the maximum SPN emissions for GDI vehicles with GPFs ranged from 1.3 to 20 with an average ratio of 10. At low idling (with or without load), GDI vehicles without GPFs emitted at least six times more SPN emissions compared to GDI vehicles with GPFs. At high idling, this ratio was higher than 16, while at loaded high idling, it was more than 20. During sharp accelerations, the ratio was 1.4, but after excluding V5, which had particularly higher SPN emissions compared to other GDI vehicles with GPFs, the ratio increased to 45. When measuring with LAB_10_, the ratio increased to 11 at low and loaded low idling, 35 at high idling, 22 at loaded high idling, and 53 during sharp accelerations (excluding V5). Note that for diesel vehicles with and without DPFs, the SPN differences are around three orders of magnitude [37].

The selection of a test protocol (or a combination of test protocols) should consider, in addition to the relative SPN concentration differences examined in the previous paragraph, the absolute SPN concentration levels, which contribute to the measurement uncertainty, especially in cases near the lower detection range of the PN-PTI instruments. At low idling, the LAB_23_ minimum difference for vehicles with and without GPFs were <10^4^ #/cm^3^ and ~10^4^ #/cm^3^ at high idling. At loaded low idling and during sharp accelerations, the minimum difference was 6.5 × 10^4^ #/cm^3^, while at loaded high idling, it was 1.3 × 10^5^ #/cm^3^. However, the SPN variation was very high and some vehicles without GPFs emitted even 10^6^ #/cm^3^ higher than vehicles with GPFs. When excluding V5, the minimum difference for V5 increased to 2.5 × 10^5^ #/cm^3^. With LAB_10_, the minimum SPN concentration differences between vehicles with and without GPFs were 2 to 5 times higher compared to LAB_23_.

PFI vehicles emitted lower SPN concentrations than GDI vehicles without GPFs. LAB_23_ at the different test protocols for three out of five vehicles (V12, V14, and V15) never exceeded 3 × 10^4^ #/cm^3^. Also, LAB_10_ concentration levels were low for these three vehicles, specifically below 8 × 10^4^ #/cm^3^ at all test protocols. V11 and V13 emitted >10^5^ #/cm^3^ at loaded high idling and during the sharp accelerations while V11 also at high idling. At low idling, only V11 emitted >10^4^ #/cm^3^ (LAB_23_ measured 2.7 × 10^4^ #/cm^3^). Interestingly, LAB_10_ emissions of V11 at low idling were even >2.5 × 10^5^ #/cm^3^.

## 4. Conclusions

Two PN measurement devices designed for diesel vehicle PN-PTI tests, one based on a diffusion charger (PTI_23_-DC) and one on a condensation particle counter (PTI_23_-CPC), were assessed with fifteen petrol cars powered by GDI and PFI engines. Two reference instruments were used, one measuring down to 23 nm (LAB_23_), similar to the PTI devices, and one measuring down to 10 nm (LAB_10_). Each vehicle underwent five different tests: low and loaded low idling, high and loaded high idling, and three sharp accelerations under static conditions. A preliminary assessment of the five different test protocols for detecting malfunctioning GPFs was also performed.

The two PTI instruments compared to LAB_23_ demonstrated very good linearity, with R^2^ values of 0.93 for PTI_23_-DC and 0.92 for PTI_23_-CPC. The slope of the PTI measurement data linear fit was close to unity, specifically 1.13 and 1.09 for PTI_23_-DC and PTI_23_-CPC, respectively. In almost all tests, the two PTI devices recorded higher concentrations than LAB_23_, and in many cases, even higher than 50%, which is the maximum error allowed in German PN-PTI regulation when measuring diesel vehicle exhaust. Sub-23 nm fractions were generally high and, in most cases, corresponded to geometric mean diameters (GMDs) ≤ 35 nm. Higher sub-23 nm fractions also resulted in higher deviations which, except in one case, occurred for vehicles without GPFs. It was not investigated whether these high sub-23 nm fractions observed at the PN-PTI tests are proportional to sub-23 nm fractions during vehicle type approval testing.

The deviations of PTI instruments compared to the reference were higher for PFI vehicles compared to GDI vehicles, possibly due to lower GMDs emitted by this engine technology. Sub-23 nm fractions for PFI vehicles averaged 60% while GDI vehicles averaged 47%. A weaker relation was found between test protocol and instrument deviations; tests performed at higher rpm (high idling, loaded high idling, and at sharp accelerations) yielded higher deviations, again possibly due to the higher sub-23 nm fraction emitted during these tests. It cannot be excluded that higher exhaust pressure at high rpm had an effect on the PN-PTI counters’ efficiency.

GDI vehicles with GPFs generally emitted lower SPN concentrations than those without GPFs and showed, in most tests, SPN concentrations below 10^4^ #/cm^3^, much lower than the diesel PN-PTI limit of 250,000 #/cm^3^. GDI vehicles without GPFs had a wide range of emissions (~10^4^–10^7^ #/cm^3^) depending on the test conditions. Some GDI vehicles without GPFs emitted higher SPN emissions at high idling and sharp accelerations while others at low idling. GDI vehicles without GPFs emitted in many cases one order of magnitude or higher SPN compared to the GPF-equipped vehicles. The differences between GDI vehicles with and without GPFs were clear but not as significant as for diesel vehicles with a well-functioning particulate filter and without a particulate filter. Loaded tests increased absolute concentration level differences. Moreover, measuring particle sizes down to 10 nm increased both relative and absolute differences. Instead, PFI vehicles without GPFs in many cases emitted low SPN emissions, near the PN-PTI instruments’ lower detection limit.

This study shows promising results regarding the PN-PTI measurement of petrol cars. However, further studies are needed with more PN-PTI instruments to examine their efficiency when measuring petrol cars as well as possible durability issues especially due to the elevated water content of gasoline vehicles’ exhaust. Lastly, additional test protocol assessments should be conducted alongside vehicle type approval cycle testing.

## Figures and Tables

**Table 1:** Main characteristics of tested vehicles.

Code	Euro	Engine	Year	Mileage (km)	Engine Displacement (cm^3^)	Power (kW)	Particulate Filter
V1	6d	GDI-Hybrid	2023	12,006	999	81	Yes
V2	6d-TEMP	GDI	2020	62,004	999	85	Yes
V3	6d	GDI	2021	47,925	1498	96	Yes
V4	6d	GDI	2022	17,325	999	92	Yes
V5	6d-TEMP	GDI	2020	25,544	1332	100	Yes
V6	5b	GDI	2012	188,435	998	49	No
V7	6b	GDI	2016	77,853	1197	81	No
V8	6b	GDI	2016	60,136	1395	92	No
V9	6b	GDI	2017	81,510	1197	85	No
V10	6b	GDI	2018	65,132	999	77	No
V11	5b	PFI	2015	172,301	1199	60	No
V12	6d-TEMP	PFI	2019	12,657	1242	66	No
V13	6b	PFI	2017	32,761	1400	70	No
V14	6b	PFI	2017	72,690	999	55	No
V15	6b	PFI	2017	32,373	1396	66	No

GDI = Gasoline Direct Injection. PFI = Port-Fuel Injection.

## Data Availability

Data are available from the corresponding authors upon request.
